# Post-Thyroidectomy Nausea and Vomiting Using Continuous Remifentanil Infusion During Emergence Depending on the Inhaled Anesthetics: A Retrospective Cohort Study

**DOI:** 10.3390/medicina62071304

**Published:** 2026-07-06

**Authors:** Ye Ji Hwang, Jeong Eun Lee

**Affiliations:** Department of Anesthesiology and Pain Medicine, School of Medicine, Kyungpook National University, Kyungpook National University Chilgok Hospital, 807, Hoguk-ro, Buk-gu, Daegu 41404, Republic of Korea; tiraremisu51@knuh.kr

**Keywords:** postoperative nausea and vomiting, remifentanil, sevoflurane, desflurane, smooth emergence

## Abstract

*Background and Objectives*: Immediately after thyroidectomy, retching driven by postoperative nausea and vomiting (PONV) may cause wound rupture, severe bleeding, and airway obstruction. Although inhaled anesthetics are widely used in thyroidectomy, they may increase the postoperative risk of PONV. Therefore, this study aimed to compare PONV incidence and recovery patterns according to the characteristics of sevoflurane (Sevo) and desflurane (Des) when remifentanil was continuously infused until extubation. *Materials and Methods*: This retrospective cohort study involved 70 female patients undergoing elective thyroidectomy, who were categorized into the Sevo (*n* = 35) and Des (*n* = 35) groups. Remifentanil was administered at an effect-site concentration of 2 ng/mL during emergence. *Results*: PONV incidence during emergence was 20% in both groups (*p* = 1.000). The Des group had shorter times to recovery of consciousness and extubation than the Sevo group (*p* < 0.001 and *p* < 0.001, respectively). At 5 min after extubation, patients in the Des group were more alert (*p* = 0.001), with 54.3% awake and responsive. Postanesthesia care unit stay was also shorter in the Des group (16.89 ± 3.22 vs. 23.74 ± 4.80; *p* < 0.001). Additionally, perioperative hemodynamic status, surgical site pain, and residual sedation did not differ between inhaled anesthetics. *Conclusions*: When remifentanil was infused until extubation after thyroidectomy, the choice of inhaled anesthetics did not affect the incidence of acute PONV. Des provided faster early recovery without additional side effects than Sevo; nonetheless, acute recovery profiles did not differ between inhaled anesthetics.

## 1. Introduction

Postoperative nausea and vomiting (PONV) is the most common complication delaying postoperative recovery, and its frequency and risk factors vary, including the type of surgery, anesthetics, and the use of opioid analgesics [1]. Thyroid surgery is known to be a surgery with a high risk of PONV, and it is presumed that vagal nerve stimulation during thyroidectomy causes a nausea or vomiting response [2]. However, the mechanism of post-thyroidectomy nausea and vomiting is still unclear, and although various antiemetic strategies have been trialed, no complete cure has been established [3].

Thyroidectomy requires surgical manipulation anterior to the trachea with the patient’s neck fully extended, and post-thyroidectomy retching associated with nausea or vomiting not only delays patient recovery but also carries the risk of causing post-thyroidectomy hemorrhage by increasing pressure at the surgical site [4]. The incidence of post-thyroidectomy bleeding reported to date is relatively low at 0.36–4.3% [5,6]. Surgical-site hematoma is a complication that requires close observation from anesthetic emergence through discharge, as it can precipitate acute airway obstruction and catastrophic events.

Although inhaled anesthetics may increase the incidence of PONV [7], they are widely used in thyroidectomy because their low solubility supports rapid induction and emergence, and precise control of anesthetic depth intraoperatively [8]. In addition, although opioids can increase PONV incidence, remifentanil is commonly infused during emergence for its central nervous system depressant effect [8,9], because excessive coughing or movement during tracheal extubation can precipitate hypertension, tachycardia, arrhythmias, myocardial infarction, bronchospasm, increased intracranial and intraocular pressure, and severe surgical-site bleeding during recovery [10,11]. Among the most commonly used inhaled anesthetics, sevoflurane (Sevo) and desflurane (Des), the effect of agent choice on PONV incidence after thyroidectomy remains controversial, and no studies have compared acute PONV in patients undergoing thyroidectomy while receiving continuous remifentanil infusion at the same concentration. Therefore, this study aimed to compare the differences in PONV according to the inhaled anesthetics during emergence with continuous remifentanil infusion. We also compared recovery profiles and complications in the postanesthesia care unit (PACU) by inhaled anesthetic.

## 2. Materials and Methods

### 2.1. Study Design and Data Collection

This study was approved by the Institutional Review Board of Kyungpook National University Chilgok Hospital. The requirement for written informed consent was waived. Data up until the discharge were manually retrieved from the patients’ medical records. This study followed the Strengthening the Reporting of Observational Studies in Epidemiology checklist [12].

This retrospective observational study was conducted at a single cancer center between September 2025 and February 2026. The study included 70 adult women (aged 24–65 years) with an American Society of Anesthesiologists physical status of I or II, scheduled for elective thyroid cancer surgery (thyroid lobectomy or total thyroidectomy without cervical lymph node dissection) under general anesthesia. Exclusion criteria comprised difficult tracheal intubation, rapid sequence induction due to a high risk of aspiration, obesity (BMI > 30 kg/m^2^), pregnancy, recent upper respiratory infection, chronic cough, current smoking, history of chronic respiratory disease (e.g., asthma or chronic obstructive pulmonary disease), or significant cardiovascular, hepatic, or renal disease. All data were anonymized prior to analysis.

### 2.2. Interventions

Anesthesia followed the standard institutional protocol and was delivered by anesthesiologists who were not involved in the study. All patients fasted for ≥8 h before surgery, and no preanesthetic medications were administered. During the surgery, patient vital signs were measured by electrocardiography, noninvasive blood pressure monitoring, pulse oximetry, esophageal temperature probe, and end-tidal carbon dioxide capnography. Depth of anesthesia was monitored by the Bispectral Index (BIS), and peripheral nerve stimulation was used to assess neuromuscular blockade. All patients were preoxygenated with 100% oxygen for ≥1 min. During anesthesia induction, propofol (2 mg/kg) was administered intravenously, and remifentanil was delivered via target-controlled infusion (TCI) at an effect-site concentration (Ce) of 2.0–6.0 ng/mL using the Minto pharmacokinetic model on the TCI device (Orchestra^®^ Base Primea; Fresenius Kabi, Sèvres, France). When the BIS dropped below 60, rocuronium (0.8 mg/kg) was administered intravenously to achieve a train-of-four (TOF) count of zero, after which a 7.0-mm internal diameter endotracheal tube was inserted using a videolaryngoscope. The endotracheal tube cuff was checked with each change in surgical position and maintained at 20–25 mmHg until extubation.

Inhaled anesthetics and remifentanil were titrated to maintain a BIS of 40–60, and were adjusted to keep the blood pressure and heart rate within 10–20% of preanesthesia values. Anesthesia was maintained with Sevo or Des at an inspired-oxygen fraction of 50% (air/O_2_) and a total gas flow of 4 L/min, with Sevo concentrations of 1.2–2.5% and Des concentrations of 3.0–8.0%. Remifentanil was administered at a Ce of 2.0–5.0 ng/mL.

Mechanical ventilation was set to a tidal volume of 8 mL/kg with a respiratory rate adjusted to maintain end-tidal carbon dioxide at 35–40 mmHg, while body temperature was maintained at 35.5–36.7 °C. During the perioperative period, Hartmann’s solution was administered intravenously via a 20-gauge cannula using an infusion pump at 100 mL/h. To maintain perioperative hemodynamic stability, intravenous glycopyrrolate (0.1 mg) was administered for bradycardia (<50 beats/min), esmolol (10 mg) for tachycardia (>110 beats/min), nicardipine (1 mg) for hypertension (mean arterial pressure > 30% above the preanesthetic value), and ephedrine (8 mg) for hypotension (mean arterial pressure drop of >20% below the preanesthetic baseline).

Both procedures were completed by two surgeons via direct closure of a 5–8 cm incision without surgical drainage. Once the thyroid specimen was removed and subcutaneous suturing had begun, remifentanil was continuously administered at a Ce of 2.0 ng/mL, and the inhaled anesthetics concentration was adjusted to 0.8 minimum alveolar concentration, achieving a BIS of 40–60 and TOF ratio of 40–90%. Sevo was maintained at 1.4–1.6%, and Des at 3.0–4.8%. After skin closure and before discontinuation of the inhaled anesthetic, ketorolac (0.5 mg/kg) was administered intravenously for analgesia. The thyroid pillow was removed and the patient placed in a neutral position. Inhaled anesthetic was then discontinued, and emergence was facilitated with 80% oxygen in air at 8 L/min. Postoperatively, the TOF ratio was monitored every 20 s. Neuromuscular blockade was reversed with intravenous sugammadex (2 mg/kg) when the TOF ratio was between 40% and 90%. Airway secretions were suctioned once with a rubber catheter via the oral airway. Ventilation was then switched to manual mode without physical stimulation until the patient opened their eyes in response to verbal command, maintaining an end-tidal carbon dioxide level of 35–44 mmHg until spontaneous breathing was restored.

Once the patient opened their eyes in response to verbal command and maintained an adequate tidal volume and respiratory rate at a TOF ratio of ≥90%, the endotracheal tube was removed, and 100% oxygen at 8 L/min was administered via face mask for 5 min. After confirming stable respiration and hemodynamics, the patient was transferred to the PACU. In the PACU, intravenous ramosetron (0.3 mg) was administered as needed for nausea or vomiting, and intravenous fentanyl (25 μg) was administered for surgical wound pain (numeric rating scale > 5).

The time to eye opening on verbal command and the time to extubation after discontinuation of the inhaled anesthetic were recorded in the electronic medical chart. Before transfer to the PACU, the level of consciousness and respiratory rate were assessed and recorded. Sedation depth was assessed by a four-point grading scale (0, deeply sedated and unresponsive; 1, sedated but responsive to light glabellar tap or loud voice; 2, sedated but responsive to normal voice; 3, awake and responsive). During emergence—from the end of surgery to PACU transfer—PONV severity was recorded on a 4-point scale (0, no PONV; 1, single retching of mild severity; 2, twice retching without gastric contents; 3, three or more retching or vomiting) [13,14]. Hemodynamic variables—mean arterial pressure and heart rate—were compared 5 min after operating room admission, at the end of surgery, at extubation, and 5 min after extubation. After PACU admission, residual sedation, hypertension, postoperative pain, nausea, and vomiting were monitored using the following criteria: hypertension, defined as a mean arterial pressure increase > 30% above baseline measured 5 min after operating room admission; pain, a numeric rating scale ≥ 5; sedation, a residual sedation score ≤ 2 at 10 min after PACU admission; nausea, the need for antiemetics without retching; and vomiting, the occurrence of retching regardless of contents. Patients were discharged from the PACU once the Aldrete score reached ≥ 9 [9].

### 2.3. Statistical Analysis

The primary endpoint was PONV incidence during emergence by inhaled anesthetic, and secondary endpoints were the occurrence of acute postoperative complications in the PACU, comprising nausea, vomiting, perioperative hypertension, postoperative wound pain, and residual sedation.

The sample size was determined from the results of our preliminary data to detect the difference in the incidence of PONV (22%), with and an α of 0.05 and a β of 0.2. A sample size of 70, 35 in each group, was calculated including potential dropouts of 10%.

Data were analyzed using IBM SPSS Statistics 22.0 (IBM Corp., Armonk, NY, USA). For normally distributed continuous variables, comparisons used an unpaired two-tailed Student’s *t*-test or repeated-measures analysis of variance with Bonferroni correction. Data were analyzed using the Mann–Whitney U test when continuous variables were not distributed normally. Categorical data were analyzed using the chi-square test or Fisher’s exact test, as appropriate. A *p*-value of 0.05 was considered statistically significant.

## 3. Results

**Figure 1:**
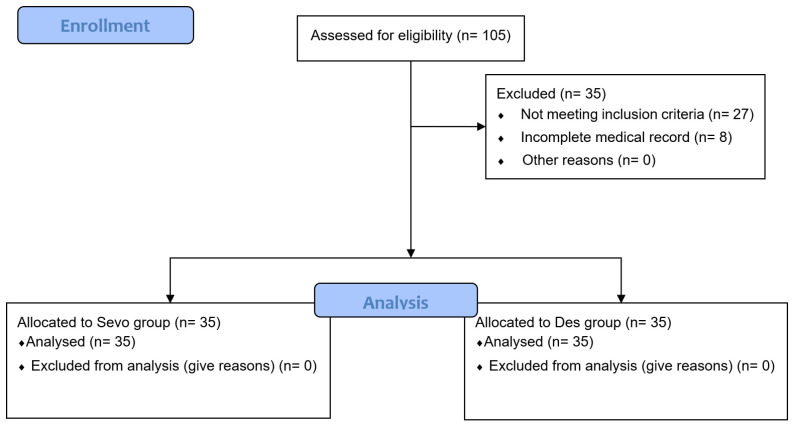
Flow diagram illustrating the enrollment process. Sevo, sevoflurane; Des, desflurane.

During the study period, 105 patients were screened; 27 met the exclusion criteria, and 8 patients were excluded due to incomplete medical records. Overall, 70 patients were included and assigned to two groups according to the inhaled anesthetic agent. No further loss to follow-up or to analysis occurred after inclusion (Figure 1).

**Figure 2:**
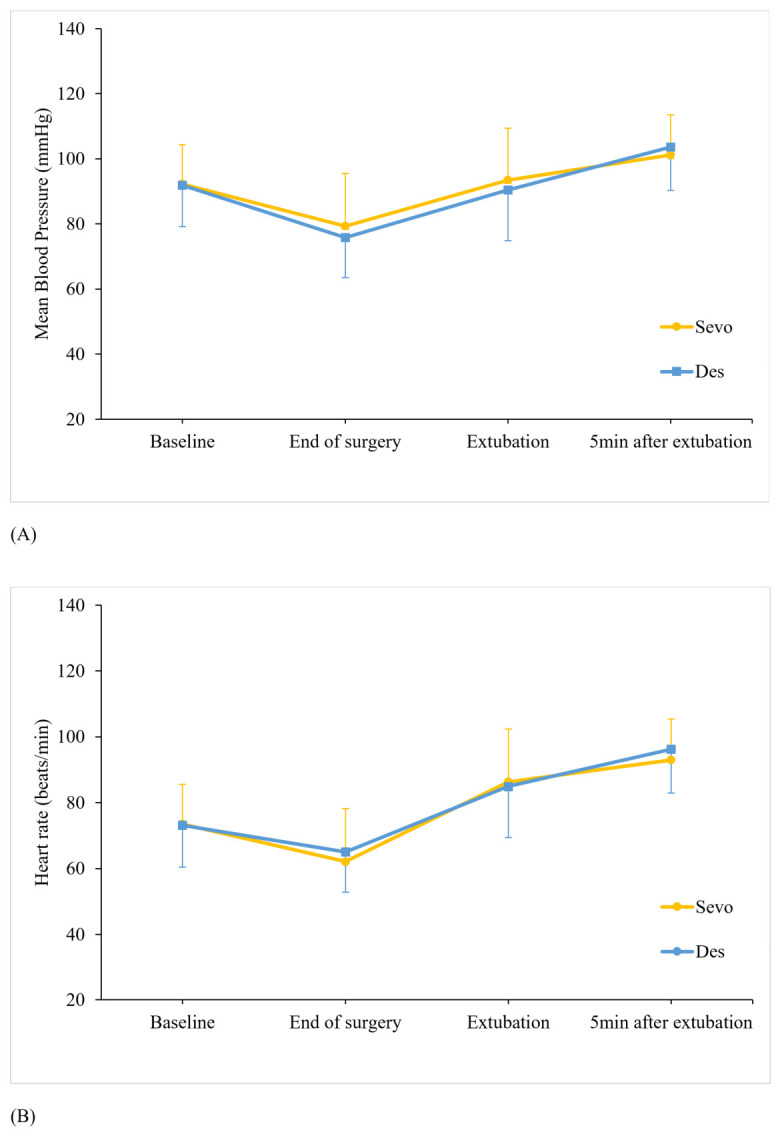
Perioperative hemodynamic status: (**A**) perioperative mean blood pressure; (**B**) perioperative heart rate.

The two groups did not differ significantly in participant physical characteristics or surgery time (Table 1). PONV incidence during emergence from anesthesia was 20.0% in both groups. PONV severity during emergence did not differ significantly between the Sevo (severe 0%, moderate 2.9%, mild 17.1%) and Des (severe 2.9%, moderate 0%, mild 17.1%) groups (Table 2). Intraoperative and acute postoperative hemodynamic variables did not differ between groups (Figure 2).

The time to eye opening on verbal command after discontinuation of the inhaled anesthetics following surgery was significantly shorter in the Des group (7.06 ± 1.95 min) than in the Sevo group (13.2 ± 3.74 min; *p* < 0.001; Table 3). Similarly, time to tracheal extubation after discontinuation of the anesthetic gas was significantly shorter in the Des group (8.69 ± 2.08 min) than in the Sevo group (15.46 ± 4.32 min; *p* < 0.001; Table 3).

Five minutes after extubation, no patients in either group were in deep sedation; however, the Des group had a slightly higher proportion of patients fully awake. Respiratory rates ranged from 11 to 17 breaths/min, with no episodes of severe bradypnea in either group and no significant between-group difference (*p* = 0.346; Table 3). PACU stay was significantly shorter in the Des group than in the Sevo group (16.89 ± 3.22 vs. 23.74 ± 4.80; *p* < 0.001; Table 4). Incidence of nausea, vomiting, severe sedation, hypertension, and tachycardia in the PACU did not differ significantly between the two groups. All patients were discharged without any surgical complications.

## 4. Discussion

With continuous remifentanil infusion through thyroidectomy, 20% of patients in both groups experienced PONV during emergence, with its severity unaffected by the inhaled anesthetic used. The Sevo group was more deeply sedated after extubation, but both groups remained alert in the PACU with no decline in consciousness. The Des group regained consciousness and respiration faster, and their PACU stay was significantly shorter than that of the Sevo group. During the PACU stay, the two groups did not differ significantly in nausea, vomiting, postoperative pain, or respiratory depression. Although numerous prospective studies have been conducted on PONV after thyroidectomy, prospective studies were limited to a highly selective patient group and controlled conditions; consequently, this retrospective was conducted without preanesthetic screening and reflected outcomes from the routine clinical application.

In this retrospective observational study, all patients had at least one Apfel risk factor as female and had medium risk for PONV [3,15,16]. Although smoking history and previous PONV history could not be verified due to the nature of the retrospective study, there has been the implementation of liberal treatment tailored to each individual rather than risk scoring for active PONV prophylaxis [17]. PONV is a clinically significant concern following thyroidectomy, particularly when inhaled anesthetics are used [18,19,20]. In thyroid surgery, PONV occurs in 60–84% of patients owing to vagal stimulation from surgical manipulation of the neck area [3,20,21,22,23]. Prospective interviews with patients after surgery show that nearly 47% of postoperative complaints were due to PONV, making it the most common patient-reported discomfort [18]. PONV delays recovery and increases the risk of aspiration and electrolyte imbalance [24]. Although all patients in this study were discharged without complications at the surgical site, retching is also one of the risk factors for post-thyroidectomy bleeding. Retching caused by PONV increases pressure at the surgical site or vascular ligation site, which can lead to wound dehiscence or hematoma, potentially resulting in reoperation or airway obstruction [25]. Here, PONV occurred in 20% of patients during emergence and 14.3% of patients in the PACU, significantly lower than that in other studies [26,27,28]. The study on thyroidectomy using opioid-based intravenous patient-controlled analgesia reported that Sevo is associated with a lower incidence of PONV than Des [29]. The higher PONV incidence in the Des group was hypothesized to result from its faster recovery of consciousness during the acute phase, which increased analgesic requirements and opioid use compared to the Sevo group [29,30]. In our study, the low incidence of PONV may reflect sustained remifentanil infusion, using only nonsteroidal anti-inflammatory drugs as analgesics before extubation, and avoiding additional opioid analgesics in the PACU. Although the Des group had clearer consciousness at extubation, no residual sedation was observed in either group in the PACU, and opioid use for severe pain (numeric rating scale ≥ 5) was comparable. Although the difference in PONV incidence between Des and Sevo remains debated, PONV incidence in the Des with remifentanil infusion was not greater than that in the Sevo group after thyroidectomy in this study [26,30,31,32].

Among the methods for smooth emergence during extubation after thyroidectomy, continuous remifentanil infusion is recognized as an effective method for cough suppression. Although prior studies have examined PONV following opioid use after recovery, the effects of sustaining remifentanil infusion through extubation remain unconfirmed. Remifentanil is characterized by rapid degradation by nonspecific esterases in blood and tissues, a low volume of distribution, and a fast elimination rate [33]. Even with continuous high-dose administration, blood drug concentration decreases rapidly, and spontaneous breathing recovers quickly once infusion stops [33]. Remifentanil suppresses cough through its central nervous system depressant effect and also reduces sympathetic tone, enabling smooth extubation without hypertension or tachycardia [8]. During Sevo anesthesia, the remifentanil Ce required to suppress cough during double-lumen endotracheal tube extubation was 1.67 ng/mL in 50% of patients and 2.28 ng/mL in 95% of patients [8]. Within our institutional protocol, a TCI of 2.0 ng/mL significantly reduced cough without delaying consciousness recovery, and this dose was used in the present elective thyroidectomy cohort. No consistent conclusion has emerged regarding PONV during continuous remifentanil infusion. Reports indicate that PONV incidence is higher when remifentanil is used in combination with inhaled anesthetics, and that PONV incidence did not differ between low and high doses under total intravenous anesthesia [34]. Continuous remifentanil infusion can cause both PONV and opioid-induced hyperalgesia [35]. Opioid analgesic consumption was hypothesized to increase during the acute recovery phase because of postoperative opioid induced hyperalgesia, leading to an elevated PONV incidence [27,36]. In a meta-analysis on perioperative remifentanil infusion [34], postoperative pain and analgesic requirement were lower in the low-dose remifentanil group within the range from 1 to 12 ng/mL. However, low-dose remifentanil produced no difference compared with control group which was not using remifentanil, and no concentration-dependent difference in PONV incidence was observed. In our cohort, a low dose of 2 ng/mL was used uniformly, but because remifentanil was maintained through extubation without dose tapering, we anticipated an effect on the acute PONV incidence. However, our results paralleled the reported PONV incidence in general surgeries, and PONV incidence in both groups was very low compared with other thyroidectomy studies [22,27]. Inhaled anesthetics increase PONV incidence in a dose-dependent manner and remain the primary risk factor for early PONV [26,27,28]. The low PONV incidence in our cohort may reflect sustained anesthesia depth via continuous remifentanil infusion through the end of surgery; the low incidence may also be attributable to the gradual reduction in inhaled anesthetics.

Studies report that the low blood–gas partition coefficient of Des promotes fast awakening and more endotracheal tube irritation was developed from emergence agitation in the absence of cough-prevention measures. In our results, at the same remifentanil concentration, sedation in the Des group remained shallow 5 min after extubation, and 54% of patients could follow instructions with full awareness. Thus, PACU stay may also be shortened. Hypertension or tachycardia from cough or pain did not differ between the groups. With proper concentration of continuous remifentanil infusion, Des may be preferable for assessing surgical complications after thyroidectomy, as it allows faster recovery of consciousness and respiration while maintaining a similar PONV incidence. Thyroidectomy carries a 4–8% risk of recurrent laryngeal nerve injury [12,37]. During surgery, the nerve may be damaged by endotracheal tube cuff compression or manual pressure when the neck is extended [38]. Inhaled anesthetics can also delay the complete reversal of neuromuscular blockade in patients after surgery. Therefore, rapid recovery from Des may reduce residual neuromuscular blockade, affecting the assessment of nerve continuity without increasing PONV incidence.

Several limitations of this study should be acknowledged. First, our focus was limited to PONV and to sedation in the operating room and PACU. Because the analysis relied on retrospective medical records, long-term recovery profiles and patient satisfaction scores could not be evaluated. Second, the surgeries in this cohort were relatively short, lasting approximately 2 h. Furthermore, since neck dissections—which involve longer surgical duration and a larger surgical field—were excluded, our results may not be generalizable. Finally, in adults, rapid consciousness recovery appears to reduce emergence agitation by allowing patients to orient to the postoperative environment [39]. In this cohort, more patients had clearer consciousness immediately after extubation with Des than with Sevo, but the degree of emergence agitation could not be ascertained from the medical records. Emergence agitation can damage the surgical site via severe neck movements or attempts to sit up as patient cooperation declines, and may exacerbate cough and wound pain from tracheal irritation. Even minor differences may influence postoperative analgesic requirement and recovery, including the risk of downstream complications. Further long-term observational studies extending this work are warranted.

## 5. Conclusions

Continuous remifentanil infusion during emergence after thyroidectomy yielded equivalent PONV incidence between Sevo and Des. Both groups also recovered comparably, with no differences in postoperative pain incidence, hemodynamic instability, respiratory complications, or residual sedation. The Des group achieved faster postoperative extubation and consciousness recovery, enabling earlier PACU discharge. Therefore, with continuous remifentanil infusion during emergence, both inhaled anesthetics can safely facilitate recovery without meaningful differences, and continuous remifentanil infusion can be used for smooth emergence without increasing PONV after thyroidectomy.

## Figures and Tables

**Table 1:** Patient demographics and duration of anesthesia by group.

	Sevo (*n* = 35)	Des (*n* = 35)	*p* -Value
Age (yr)	45.34 ± 9.41	45.23 ± 9.68	0.96
ASA physical status (n)			0.235
I	30 (85.7)	26 (74.3)	
II	5 (14.3)	9 (25.7)	
Height (cm)	157.5 ± 4.66	159.60 ± 4.74	0.066
Weight (kg)	60.79 ± 7.46	62.65 ± 9.79	0.372
Operation time (min)	105.4 ± 22.49	107.34 ± 24.12	0.729

Values are mean ± standard deviation or number (%). Sevo, sevoflurane; Des, desflurane; ASA, American Society of Anesthesiologists.

**Table 2:** Postoperative nausea and vomiting incidence during emergence.

	Sevo (*n* = 35)	Des (*n* = 35)	*p* -Value
PONV incidence (n)	7 (20)	7 (20)	1.000
Severity (n)			0.993
No PONV	28 (80.0)	28 (80.0)	
Mild (Single retching with mild severity)	6 (17.1)	6 (17.1)	
Moderate (Twice retching without gastric contents)	1 (2.9)	0	
Severe (Three or more retching or vomiting)	0	1 (2.9)	

Values are number (%). PONV, postoperative nausea and vomiting; Sevo, sevoflurane; Des, desflurane.

**Table 3:** Awakening variables during emergence.

	Sevo (*n* = 35)	Des (*n* = 35)	*p* -Value
From the end of surgery to eye opening (min)	13.2 ± 3.74	7.06 ± 1.95	<0.001
From the end of inhalation agent to extubation (min)	15.46 ± 4.32	8.69 ± 2.08	<0.001
Depth of sedation after extubation			<0.001
Deeply sedated and unresponsive	0	0	
Sedated but responsive to light glabellar tap or loud voice	11 (31.4)	1 (2.9)	
Sedated but responsive to normal voice	8 (22.9)	15 (42.9)	
Awake and responsive	16 (45.7)	19 (54.3)	
Respiratory rate after extubation (breaths/min)	14.49 ± 3.34	13.8 ± 2.68	0.346

Values are mean ± standard deviation or number (%). Sevo, sevoflurane; Des, desflurane.

**Table 4:** Recovery profiles at the postanesthesia care unit.

	Sevo (*n* = 35)	Des (*n* = 35)	*p* -Value
PACU stay (min)	23.74 ± 4.80	16.89 ± 3.22	<0.001
Nausea (n)	5 (14.3)	5 (14.3)	1.000
Vomiting (n)	0	0	1.000
Deep sedation (n)	0	0	1.000
Hypertension or tachycardia (n)	1 (2.9)	3 (8.6)	0.614
Wound pain (n)	6 (17.1)	10 (28.6)	0.255

Values are mean ± standard deviation or number (%). Sevo, sevoflurane; Des, desflurane; PACU, postanesthesia care unit.

## Data Availability

The datasets generated or analyzed during the current study are available from the corresponding author on reasonable request.

## Abbreviations

The following abbreviations are used in this manuscript:

PONV	Post Operative Nausea and Vomiting
Sevo	Sevoflurane
Des	Desflurane
PACU	Post Anesthesia Care Unit
BIS	BIspectral Index
TCI	Target-Controlled Infusion
